# Supplementary material for: Widespread alterations in microRNA biogenesis in human Huntington’s disease putamen
Source: Acta Neuropathol Commun. 2022 Jul 22;10:106. doi: 10.1186/s40478-022-01407-7 (PMC9308264; doi:10.1186/s40478-022-01407-7)
Supplement: Supplementary file 1 — Additional file 1. Supplementary Methods (Human blood samples). [file 40478_2022_1407_MOESM1_ESM.pdf]

Supplementary Methods

Human blood samples

All work with human subjects was approved by the CHU de Québec human ethics committee (#2020-4622) and in accordance with the Declaration of Helsinki. This cohort was recruited from the Clinique des troubles du mouvement of the CHU de Québec, Québec City, Canada. Informed written consent was obtained from all participants. A total quantity of 4 ml of blood was collected from patients with HD at all stages of disease (n=8) along with healthy control subjects (n=7) for a total of 15 participants. Clinical evaluations were conducted on the same day of blood sampling. Whole blood: Total RNA was isolated from 500 µl of whole blood using the TRIzol reagent (Ambion by Life Technologies) according to the manufacturer's instructions. PBMCs: peripheral blood mononuclear cells (PBMCs) were first isolated from 3 ml of whole blood using Ficoll-Plaque PLUS (GE Healthcare, 71-7167-00 AG) according to manufacturer's instruction. Then, purification of total RNA was extracted from PBMCs using TRIzol reagent (Ambion by Life Technologies) according to the manufacturer's instructions. RNA was then stored at -80°C until use. Additional details are available upon demand.
