# Supplementary material for: Widespread alterations in microRNA biogenesis in human Huntington’s disease putamen
Source: Acta Neuropathol Commun. 2022 Jul 22;10:106. doi: 10.1186/s40478-022-01407-7 (PMC9308264; doi:10.1186/s40478-022-01407-7)
Supplement: Supplementary file 2 — Additional file 2. Supplementary Tables (List of antibodies). [file 40478_2022_1407_MOESM2_ESM.pdf]

Supplementary Tables

Table S1. Antibodies used in this study.

	Antibody	Provider	Cat number	Species	Dilution	Proteins	System
Huntingtin	Anti-Htt (CHU00146)	Coriell Institute	CHDI-90000137	Rabbit	1/1000	20 µg	Tris-acetate 3-15%
Htt total	Anti-Htt (1HU-4C8)	Millipore	mab2166	Mouse	1/1000	-	Dot blot
Htt poly-Q	Anti-polyglutamine expansion (5TF1-1C2)	Millipore	mab1574	Mouse	1/1000	-	Dot blot
NeuN	Anti-NeuN (E4M5P)	Cell Signaling Technology	94403	Mouse	1/10 000	5 µg	10% acrylamide
Darpp32	Anti-DARPP-32 (19A3)	Cell Signaling Technology	2306	Rabbit	1/10 000	5 µg	10% acrylamide
PSD95	Anti-PSD95	Cell Signaling Technology	2507	Rabbit	1/1000	10 µg	10% acrylamide
DGCR8	Anti-DGCR8 [EPR18757]	Abcam	ab191875	Rabbit	1/1000	10 µg	10% acrylamide
TRBP	Anti-TRBP [EPR13550]	Abcam	ab180947	Rabbit	1/1000	10 µg	10% acrylamide
Exportin5	Anti-Exportin 5 (D7W6W)	Cell Signaling Technology	12565	Rabbit	1/1000	10 µg	10% acrylamide
Dicer	Anti-Dicer (F-10)	Santa Cruz Biotechnology	sc-136979	Mouse	1/1000	10 µg	10% acrylamide
Drosha	Anti-Drosha (D28B1)	Cell Signaling Technology	3364	Rabbit	1/1000	10 µg	10% acrylamide
Ago2	Anti-Argonaute 2 (C34C6)	Cell Signaling Technology	2897	Rabbit	1/1000	10 µg	10% acrylamide
ATG9a	Anti-Atg9a (D4O9D)	Cell Signaling Technology	13509	Rabbit	1/1000	10 µg	10% acrylamide
P62	Anti-SQSTM1/p62	Cell Signaling Technology	5114	Rabbit	1/1000	10 µg	10% acrylamide
LC3	Anti-LC3B	Novus Biological	NB 100-2220	Rabbit	1/1000	20 µg	Tris-acetate 3-15%
Beclin	Anti-Autophagy pack	Novus Biological	NB 910-94877	Rabbit	1/1000	10 µg	10% acrylamide
ATG5	Anti-ATG5	Novus Biological	NB 110-53818	Rabbit	1/1000	10 µg	10% acrylamide
TDP43	Anti-Tdp43 (3H8)	Millipore	mabN45	Rabbit	1/1000	10 µg	10% acrylamide
