# Supplementary material for: Widespread alterations in microRNA biogenesis in human Huntington’s disease putamen
Source: Acta Neuropathol Commun. 2022 Jul 22;10:106. doi: 10.1186/s40478-022-01407-7 (PMC9308264; doi:10.1186/s40478-022-01407-7)
Supplement: Supplementary file 3 — Additional file 3. Supplementary Figures (Comparative analysis of protein expression between brain regions; Analysis of mRNA expression in HD brain; miRNA screening in HD brain; qRT-PCR analysis of normalization genes; Analysis of miRNA maturation in blood). [file 40478_2022_1407_MOESM3_ESM.pdf]

Supplementary Figures

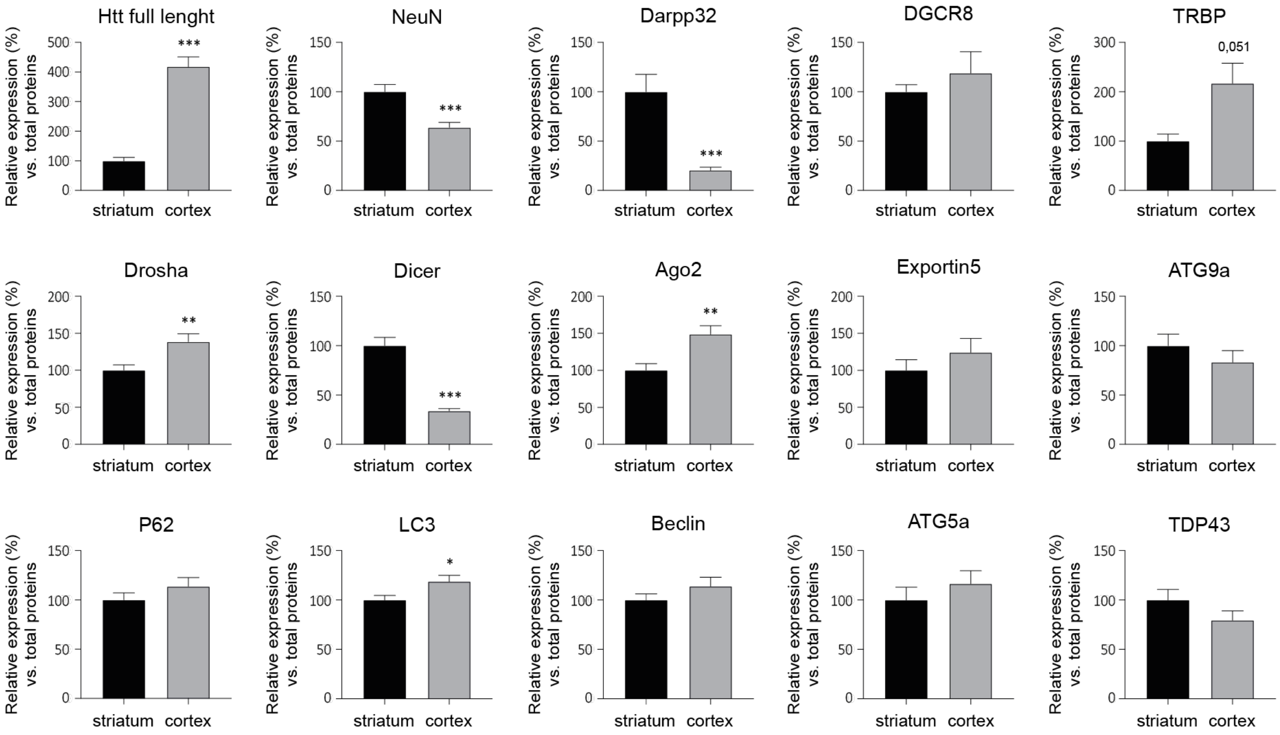

Figure S1. Comparative analysis of protein expression between brain regions. Shown here are Western blot quantifications of different proteins tested in this study. Soluble proteins extracted from the striatum and the cortex (N=25 healthy controls) were loaded onto the same gel for a side-by-side comparison. Bar graphs with standard error of the mean (SEM) are shown, where the average of the striatum is set as 100%. Statistics: Striatum vs. Cortex was calculated using a Mann-Whitney test. * $P<0.05$; ** $P<0.01$; *** $P<0.001$; **** $P<0.0001$. Trends are shown as well.

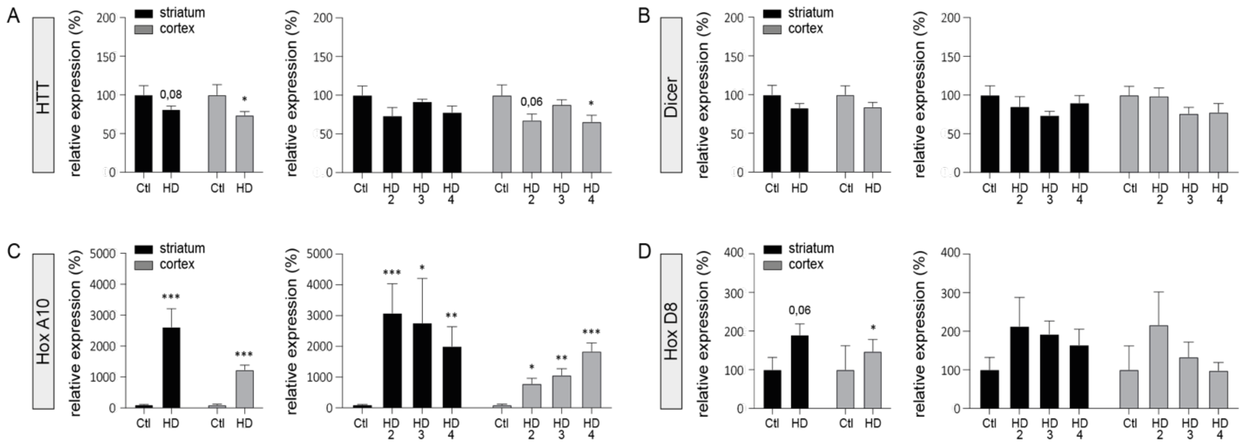

Figure S2. Analysis of mRNA expression in HD brain. qRT-PCR analysis of endogenous Htt, Dicer, HoxA10 and HoxD8 mRNA in HD patients (HD2 N=9; HD3 N=9 and HD4 N=8)) and healthy controls (N=9). Statistics: Ctl vs. HD as a group was calculated using a Mann-Whitney test. Ctl vs. HD stages was calculated using an analysis of covariance followed by Kruskal-Wallis multiple comparison test. Significant fold changes are provided for each group. * $P<0.05$; ** $P<0.01$; *** $P<0.001$; **** $P<0.0001$. Trends are shown as well. Abbreviations: Ctl, Controls; HD, Huntington's disease; HD2, Vonsattel grade 2; HD3, Vonsattel grade 3; HD4, Vonsattel grade 4.

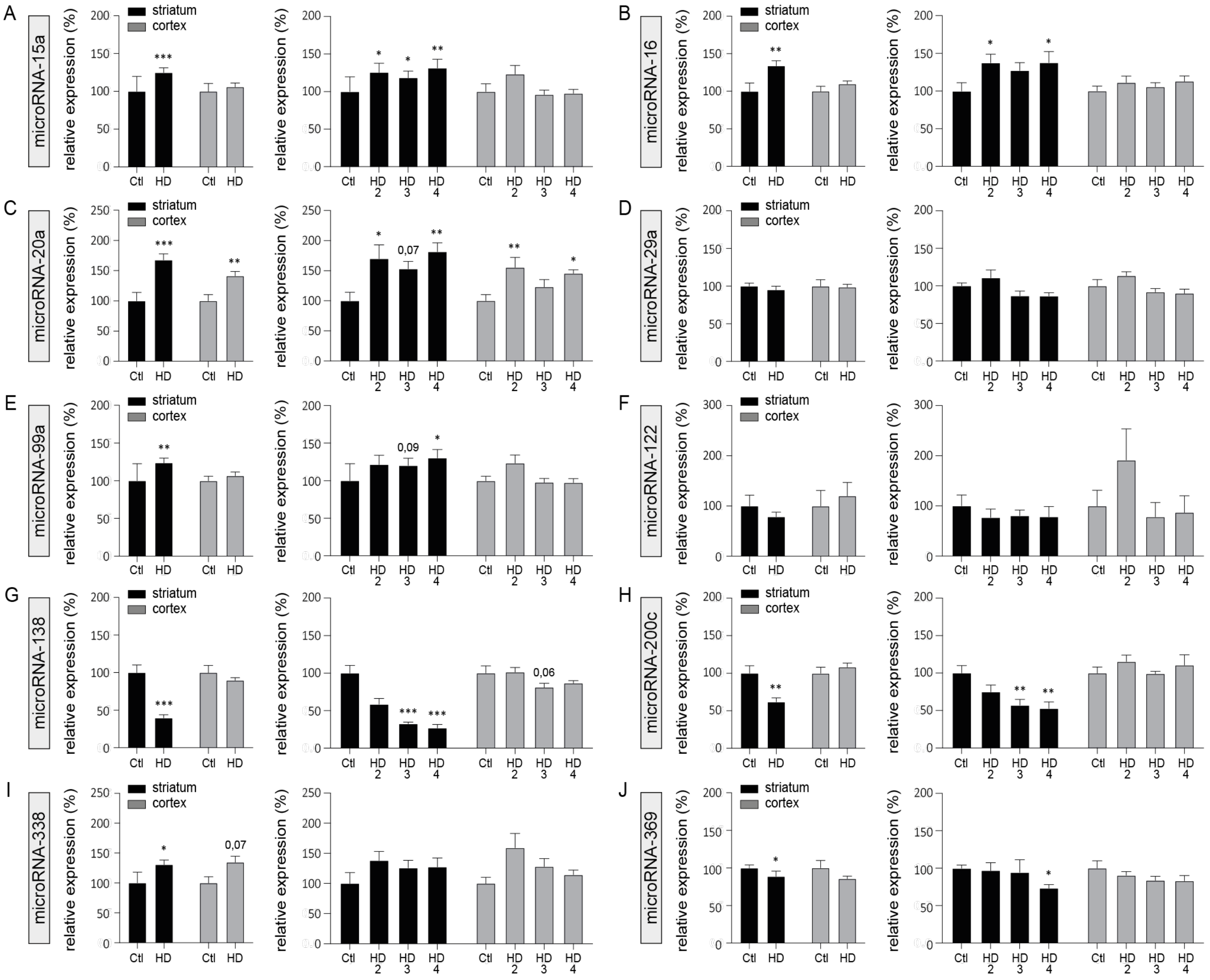

Figure S3. miRNA screening in HD brain. The relative expression levels of additional HD-related mature miRNAs were evaluated by miRNA qRT-PCR in HD patients (HD2 N=9; HD3 N=9 and HD4 N=8) and healthy controls (N=9). Most changes occurred in the striatum. Statistics: Ctl vs. HD as a group was calculated using a Mann-Whitney test. Ctl vs. HD stages was calculated using an analysis of covariance followed by Kruskal-Wallis multiple comparison test. Significant fold changes are provided for each group. * $P<0.05$; ** $P<0.01$; *** $P<0.001$; **** $P<0.0001$. Trends are shown as well. Abbreviations: Ctl, Controls; HD, Huntington's disease; HD2, Vonsattel grade 2; HD3, Vonsattel grade 3; HD4, Vonsattel grade 4.

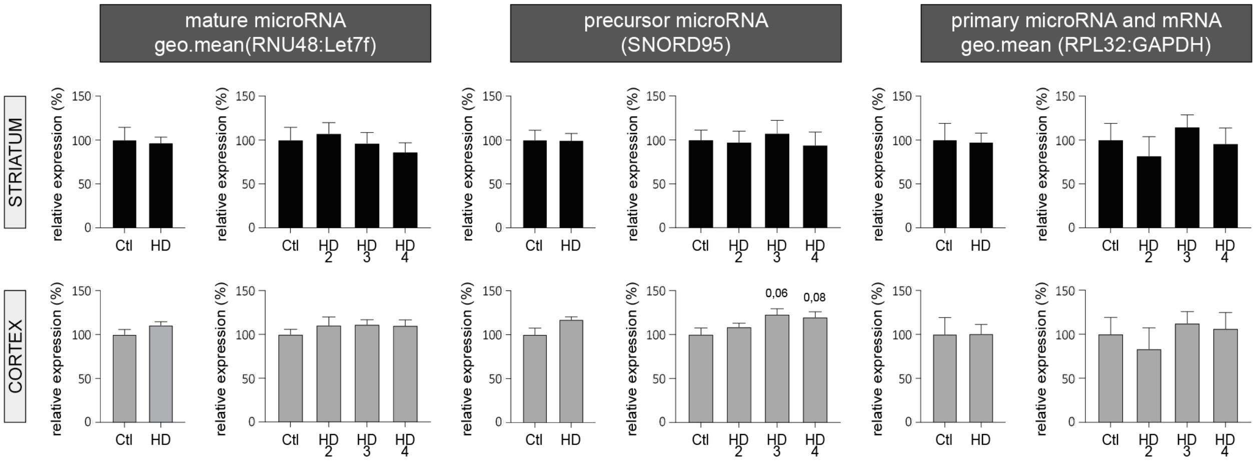

Figure S4. qRT-PCR analysis of normalization genes used in this study. Quantifications were done using both HD patients (HD2 N=9; HD3 N=9 and HD4 N=8) and healthy controls (N=9). Statistics: Ctl vs. HD as a group was calculated using a Mann-Whitney test. Ctl vs. HD stages was calculated using an analysis of covariance followed by Kruskal-Wallis multiple comparison test. No statistically significant differences were observed overall. Abbreviations: Ctl, Controls; HD, Huntington's disease; HD2, Vonsattel grade 2; HD3, Vonsattel grade 3; HD4, Vonsattel grade 4.

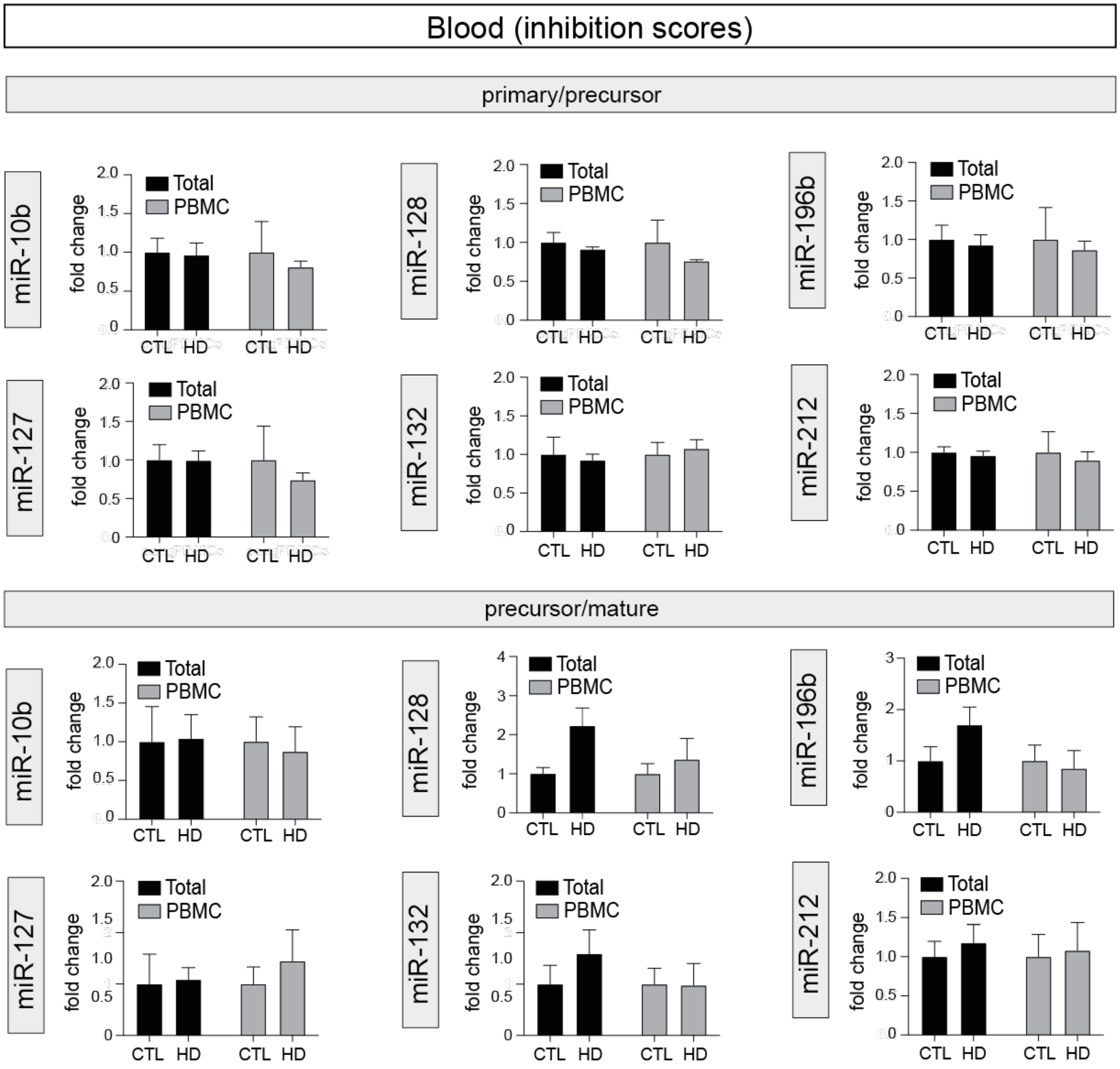

Figure S5. Analysis of miRNA maturation in blood. Overview of miRNA inhibition scores in human blood of HD (N=7) and healthy control (N=8) subjects of the CHU de Québec cohort. Here, we used total RNA extracted from either whole blood or PBMCs. No significant effects were observed for all tested miRNAs. In addition, no changes in mature miRNA levels were noted in these samples (not shown). Statistics: Ctl vs. HD as a group was calculated using an ANOVA test with multiple comparisons.
